# Short-Chain Fatty Acids Calibrate RARα Activity Regulating Food Sensitization

**DOI:** 10.3389/fimmu.2021.737658

**Published:** 2021-10-14

**Authors:** Xiefang Yuan, Hongmei Tang, Renlan Wu, Xingjie Li, Hongyu Jiang, Zhigang Liu, Zongde Zhang

**Affiliations:** ^1^ Inflammation & Allergic Diseases Research Unit, Affiliated Hospital of Southwest Medical University, Luzhou, China; ^2^ The School of Basic Medical Sciences, Southwest Medical University, Luzhou, China; ^3^ State Key Laboratory of Respiratory Disease for Allergy at Shenzhen University, Shenzhen University School of Medicine, Shenzhen, China

**Keywords:** allergy, dendritic cell (DC), microbiota, short-chain fatty acids (SCFAs), type I interferon (IFN-I)

## Abstract

Gut-microbiota dysbiosis links to allergic diseases. The mechanism of the exacerbation of food allergy caused by gut-microbiota dysbiosis remains unknown. Regulation of retinoic acid receptor alpha (RARα) signaling is critical for gut immune homeostasis. Here we clarified that RARα in dendritic cells (DCs) promotes Th2 cell differentiation. Antibiotics treatment stimulates retinoic acid signaling in mucosal DCs. We found microbiota metabolites short-chain fatty acids (SCFAs) maintain IGF-1 levels in serum and mesenteric lymph nodes. The IGF-1/Akt pathway is essential for regulating the transcription of genes targeted by RARα. And RARα in DCs affects type I interferon (IFN-I) responses through regulating transcription of IFN-α. Our study identifies SCFAs crosstalk with RARα in dendritic cells as a critical modulator that plays a core role in promoting Th2 cells differentiation at a state of modified/disturbed microbiome.

## Introduction

Vitamin A plays a crucial role in maintaining homeostasis at the intestinal barrier and balancing immunity and tolerance. As its principal active metabolite, retinoic acid (RA) implicates diverse inflammatory responses, which affect innate and adaptive immunity (1). RA involved in immunological procession regulates gene expression through binding several families nuclear hormone receptor, including retinoic acid receptors (RARs) α, β, and γ, retinoid X receptors (RXRs) α, β and γ, and the peroxisome proliferator-activated receptors (PPARs) β, δ (2). The RAR family includes RARα, RARβ, and RARγ. Lack of RA associates with abnormal migration of immune cells to the intestine and impaired immune tolerance (3, 4). RARα is the dominant retinoic acid signaling transcription factor in DCs (5). However, whether RARα in DCs regulates immune response, especially the T cell differentiation, is not clear.

RA signaling implicates the imbalance of gut immune maintenance caused by microbiota dysbiosis (6). And microbiota colonized has played a crucial role in developing the intestinal immune system (7). Microbial components and metabolites produced by gut microbiota participate in various host processes (8, 9). Short-chain fatty acids (SCFAs) are the most studied microbial metabolites produced by gut flora through the fermentation of polysaccharides. SCFAs associates with the maturation of the immune system, including induction of peripheral regulatory T cells, protection from infection, and modulation of metabolic rate and energy homeostasis (7).

Allergic diseases exert a devastating global impact and lack effective vaccines or advanced therapeutics. Allergic inflammation is a type 2 immune disorder classically characterized by the high level of immunoglobulin E (IgE) and the development of Th2 cells (10). Basic understanding of the critical cell types and mediators that initiate and modulate type 2 immunity is limited. Recently, type I interferons (IFN-I) were shown to cause Th2-cells differentiation (11). IFN-I is most well-known for its pro-inflammatory role in antiviral immunity. Many IFN-I effects are mediated by a direct impact on DC phenotype and functionality. IFN-I responsiveness controls the ability of cDC1s to present viral antigens to CD8+ T cells (12) and influences DC activation, migration, and T cell priming *in vitro*. DC-intrinsic IFN-I signaling is required for their effective migration, localization, and Th2 response *in vivo* (13).

This study showed that RARα in DCs was involved in the microbiota dysbiosis-induced exacerbation of food allergy. Loss of dendritic cells RARα repressed Th2-cell differentiation. Gut dysbiosis caused a reduction of IGF-1 expression in mLN tissue and induced hyperresponsiveness of RA signaling and IFN-I response in mLN. And the IGF-1/Akt pathway has been implicated in the suppression of RA signaling and IFN-I response by regulating RARα transcriptional ability *in vivo*. These findings identify that RARα serves as a regulatory node in food allergy. The IGF-1/Akt pathway inhibits RA signaling and IFN-I response, which played a positive role in Th2-cell differentiation.

## Results

### Retinoic Acid Signaling Is Required for Gut Microbial Dysbiosis-Induced Allergy

**Figure 1:**
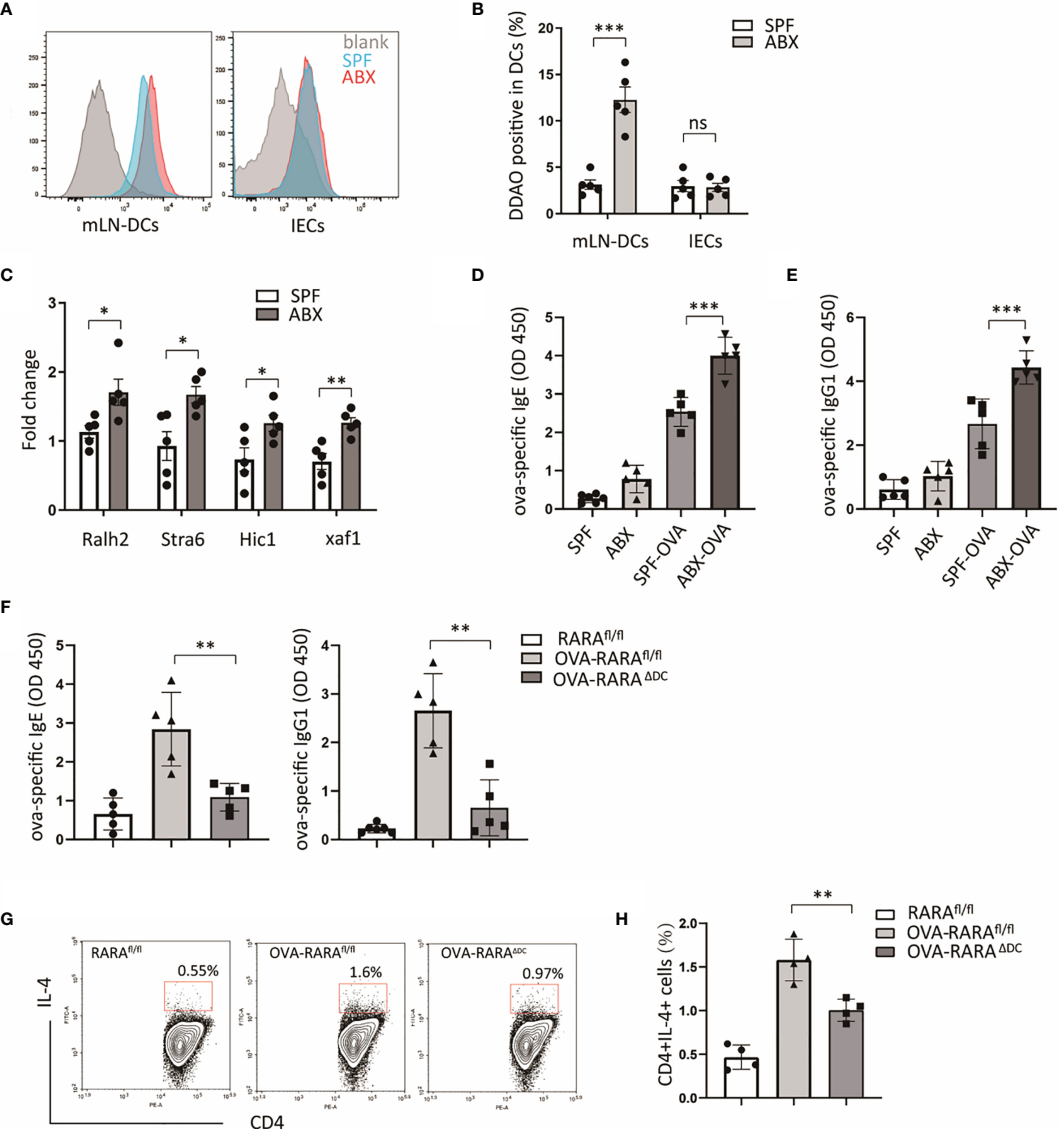
RA signaling is required for gut dysbiosis-induced allergy. **(A, B)** RA signaling assessment. β-galactosidase expression was detected by its substrate DDAOG using flow cytometry. DCs (gating on cd11c positive) from mLN tissue and intestinal epithelial cells (IECs) isolated from RARE-lacZ transgenic mice were analyzed. **(C)** RT-PCR measurement of retinoic acid signaling-related genes, relative to Gadph, in mLN tissue. **(D–F)** RARα^fl/fl^ and RARα^ΔDC^ mice, after treated with ova intragastric administration, ova-specific IgE and IgG1 levels in serum were detected by ELISA. And the frequency of CD4(+)IL4(+) cells in mLN were measured by flow cytometric analysis. Graph shows mean and SEM. Five mice per group from at least two independent experiment. Data were analyzed by unpaired Student’s t-test **(A–C)** and one-way ANOVA **(D–H)**. *p < 0.05, **p < 0.01, ***p < 0.001, ns, not significant.

Intestinal epithelial and dendritic cells are the main sites of retinoic acid metabolism in the intestinal tissues (14, 15). Specific intestinal microbiota can modulate retinoic acid signaling in intestinal epithelial cells and DCs (16, 17). To assess whether gut bacteria have an effect on retinoic acid (RA) signaling, we established an animal model with gut microbiota dysbiosis by treating mice with a cocktail of antibiotics, and RA signaling reporter mice which harbored a RA response element (RARE) upstream of β-galactosidase (LacZ) were used. The LacZ expression in epithelial and dendritic cells in mesenteric lymph nodes (mLN) was measured by flow cytometry. Then we found that mice treated with antibiotics preserved a higher level of RARE transcription in dendritic cells in mLN (**Figures 1A, B**). However, ablation of gut bacteria have no effect on RARE transcription level in intestinal epithelium (**Figures 1A, B**). Furthermore, we observed a higher expression of several RA-responsive genes in mesenteric tissue after antibiotics treatment (**Figure 1C**) using real-time quantitative PCR. These results indicate that gut bacterial regulation of RA signaling was specific in intestinal dendritic cells.

To explore whether partially depletion of gut microbiota could exacerbate food allergy, an animal model allergic to OVA was build. Consistent with other animal experiments (18), a higher level of OVA-specific IgE, IgG1 were observed in ABX treated mouse compared to untreated mouse (**Figures 1D, E**). We hypothesized that RA signaling was involved in antibiotic-induced food allergy.

To evaluate the RA signaling impact on allergic intestinal inflammation, we generated a cell-specific loss-of-function mouse model of RARα. We generated cd11c^cre^RARα^fl/fl^ mice that lack RARα in cd11c positive dendritic cells (RARA^ΔDC^). The number of CD4(+) and CD8(+)T cells in the spleen did not be affected by the loss of RARα in dendritic cells in our previous study (19). In an OVA-allergic animal model, we observed that a decreasing number of CD4(+)IL4(+) cells in RARA^ΔDC^ mice compared to RARA^fl/fl^ mice (**Figure 1G**), indicating that RARα in dendritic cells plays an essential role in Th2 cell differentiation.

### Gut Dysbiosis Regulates the IGF-1/Akt Pathway in Mesenteric Lymph Tissue

**Figure 2:**
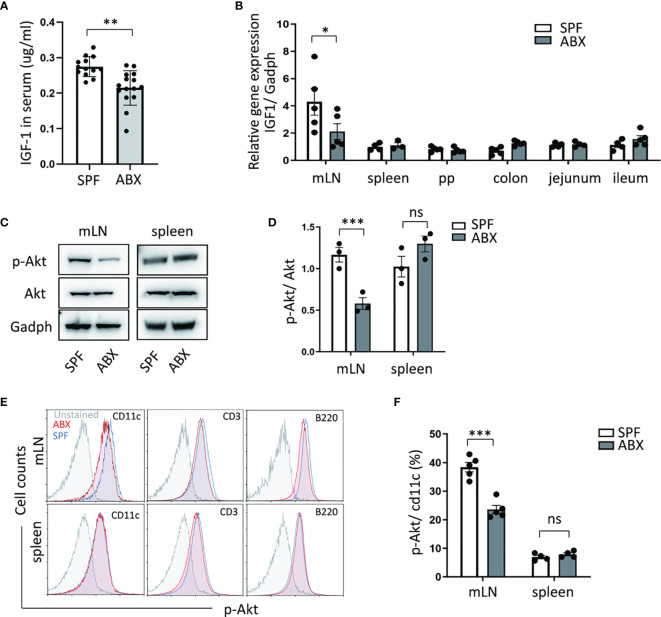
Gut dysbiosis regulates the IGF-1/Akt pathway in mesenteric lymph tissue. **(A)** After mice treated with ABX, IGF-1 ELISA was performed on serum. **(B)** And mRNA expression of IGF-1 in mLN, spleen, Peyer’s Patches (PP), colon, jejunum, ileum tissues, were measured by RTPCR. **(C, D)** p-Akt and Akt protein level change in mLN and spleen were determined by western blotting. **(E)** p-Akt expression levels of DCs (cd11c+), T cells (CD3+), B cells (B220+) in mLN and spleen were measured by flow cytometry. **(F)** Graph shows frequency of p-Akt positive DCs in mLN and spleen. As shown, p-Akt protein level of DC in mLN was reduced in ABX mice. Graph shows mean and SEM. **(A)** Data are combined from two independent experiments with at least thirteen mice per group. Graph shows mean and SEM. Data were analyzed by unpaired Student’s t-test. *p < 0.05, **p < 0.01, ***p < 0.001, ns, not significant.

Gut microbiotas inhabit the gut and affect host physiology. Gut microbiota dysbiosis, which could be induced by antibiotics abuse or microbial environmental change, has been involved in several disease processes. A recent study identified the role of gut microbiota in the regulation of bone growth by affecting circulating IGF-1 levels (20). We confirmed that the reduction of circulating IGF-1 production in ABX mice compared to untreated mice (**Figure 2A**). qPCR analysis revealed that intestinal bacteria ablation diminished the IGF-1 expression in mLN tissue. However, we did not detect IGF-1 expression differences in Peyer’s patches, spleen, and intestinal tissues, including colon, jejunum, ileum. (**Figure 2B**).

Meanwhile, we measured the protein level of Akt and its phosphorylation level, one of the most crucial molecule targets of IGF-1. We found a reduction of Akt phosphorylation level in the mesenteric lymph node after antibiotic depletion of gut microbiota coincided with decreased IGF-1 production in mLN, while the Akt phosphorylation level did not change in the spleen (**Figures 2C, D**). Moreover, we detected the Akt phosphorylation level in dendritic cells, T cells, and B cells in mLN and spleen tissue. We observed that in comparison to untreated mice, ABX mice had a decreased level of Akt phosphorylation in mLN dendritic cells (**Figures 2E, F**). These data suggest that gut bacteria regulates IGF-1 production to modulate Akt phosphorylation in dendritic cells specifically in mLN.

### IGF-1/Akt Pathway Inhibits the RA Response Through Enhancing RARA Phosphorylation

**Figure 3:**
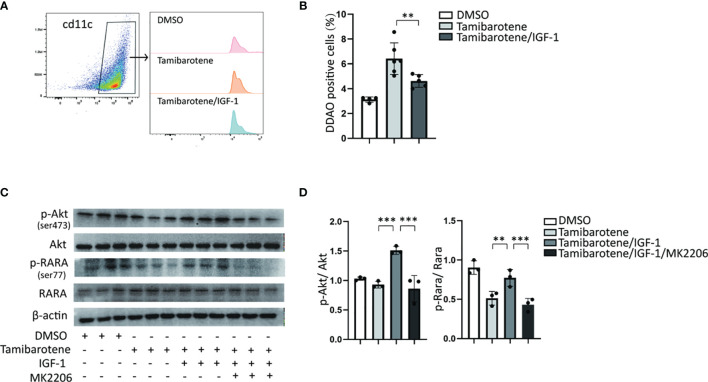
IGF-1/Akt pathway inhibits the RA response through enhancing RARA phosphorylation. **(A, B)** RA signaling assessment. BMDCs cultured from RARE-lacZ transgenic mice were treated with DMSO, Tamibarotene (4mM) alone or with IGF-1 (500ng/ml).DDAO signaling were measured by flow cytometry after BMDC treated with substrate DDAOG. **(C, D)** BMDCs were cultured from normal SPF mice, and treated with DMSO, Tamibarotene, IGF-1 and Akt inhibitor (MK2206), then p-Akt, Akt, p-RARα, RARα expression were detected by western blotting. Data are from two or three independent experiments. Graph shows mean and SEM. Data were analyzed by one-way ANOVA. **p < 0.01, ***p < 0.001.

RARα activates transcription of retinoic acid response-related genes through its dimerization with RXRa. And RARα transactivation is regulated by its Phosphorylation. RARs have been reported to be substrates for PKA (protein kinase A), PKC. Akt, a serine/threonine kinase, phosphorylates RARα to reduce its transactivation ability in NSCLC cells (21). Next, we wanted to assess whether IGF-1 modulated RARα transactivation by regulation of Akt phosphorylation. We used tamibarotene, a RARα specific agonist, to induce RA response. *In vitro* experiment demonstrated that IGF-1 inhibits RARE transcription level stimulated by tamibarotene in BMDC (**Figures 3A, B**). In the western blot experiment, tamibarotene significantly reduced the phosphorylation of RARα in BMDCs. And we found that IGF-1 increased Akt phosphorylation level, and also RARα phosphorylation level in BMDCs compared to BMDCs stimulated with tamibarotene alone. (**Figures 3C, D**). Furthermore, MK2206, an Akt phosphorylation inhibitor, suppressed the Akt phosphorylation level induced by IGF-1 and reduced RARα phosphorylation level caused by IGF-1 (**Figures 3C, D**). These results establish that IGF-1 increases RARα phosphorylation through the Akt pathway to reduce RARα transactivation ability.

### Retinoic Acid Receptor Alpha Regulates Type I Interferons and stat1 Expression

**Figure 4:**
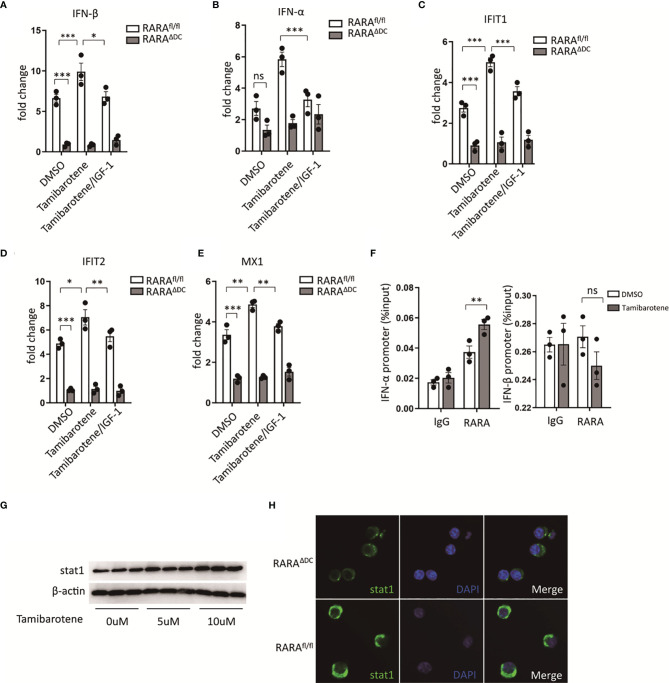
Retinoic acid receptor alpha regulates type I interferons and stat1 expression. **(A–E)** BMDCs cultured from RARα^fl/fl^ and RARα^ΔDC^ mice, were treated with DMSO, Tamibarotene (4mM) alone or with IGF-1 (500ng/ml), then mRNA expression of IFN-α, IFN-β, and ISGs (IFIT1, IFIT2, MX1) were determined by RT-PCR. **(F)** ChIP-qPCR for RARα at IFN-α, and IFN-β regulatory element in BMDCs cultured from SPF mice. **(G)** BMDCs from SPF mice were treated with different concentration of tamibarotene (respectively 0uM, 5uM, 10uM), and stat1 protein expression were measured by western blotting. **(H)** stat1 expression in BMDCs from RARα^fl/fl^ and RARα^ΔDC^ mice were detected by immunofluorescence method. Data are from at least two independent experiments. Graph shows mean and SEM. Data were analyzed by one-way ANOVA. *p < 0.05, **p < 0.01, ***p < 0.001. ns, not significant.

Type 2 immunity plays a crucial role in classic allergic diseases. Despite inflammatory mediators, cytokines, or innate immune cells influence type 2 immunity, and DCs must affect activation and polarization of type 2 immunity (22). IFN-I is well known for its antiviral immunity. However, several studies provide evidence that IFN-I signaling enhances the ability to polarize the type 2 immunity (23, 24). By *in vitro* experiment, the expression of IFN-I related genes was assessed through qPCR after stimulation of tamibarotene, a RARα specific agonist. The expression of IFN-α and IFN-β (**Figures 4A, B**) in BMDCs, the main subtypes of IFN-I, was increased after tamibarotene stimulation. Interestingly, IGF-1 could interfered with tamibarotene’s ability to exacerbate IFN-I expression in BMDC (**Figures 4A–E**). Further, to determine whether RARα affects IFN-I signaling, BMDC lacking RARα expression generated from cd11c^cre^RARα^fl/fl^ (RARA^ΔDC^) mice were used for experiments. The mRNA expression of IFN-α, IFN-β were significantly down-regulation in BMDC from RARA^ΔDC^ mice compared to RARA^fl/fl^ mice (**Figures 4A, B**). In addition, the IFN-stimulated genes (ISGs) IFIT1, IFIT2, and MX1, were also upregulated by treating tamibarotene and restricted by IGF-1 and RARα expression (**Figures 4A–E**).

We also checked by chromatin immunoprecipitation (ChIP) whether RARα binds to the promoter region of IFN-α, IFN-β to investigate the mechanism of how RARα regulates IFN-I signaling. We confirmed that RARα was bond to the promotor region of IFN-α, but not IFN-β (**Figure 4F**). Furthermore, we detected significantly higher enrichment of RARα in IFN-α binding sites in BMDC treated with tamibarotene compared with PBS-treated BMDC (**Figure 4F**).

Previous reports indicate that RARα regulates stat1 expression through the binding RARE site in stat1 promoter (25, 26). We tested the stat1 protein level after BMDC was treated with tamibarotene and found that tamibarotene increased stat1 protein expression (**Figure 4G**). The reduction of Stat1 expression in BMDC from RARA^ΔDC^ mice compared to RARA^fl/fl^ mice was observed by the immunofluorescence method (**Figure 4H**). Taken together, the results indicate that RARα in DCs regulate type I interferons signaling by modulating transcription of IFN-α.

### IGF-1 Suppresses Th2 Differentiation Instructed by RARα-Activated BMDC

**Figure 5:**
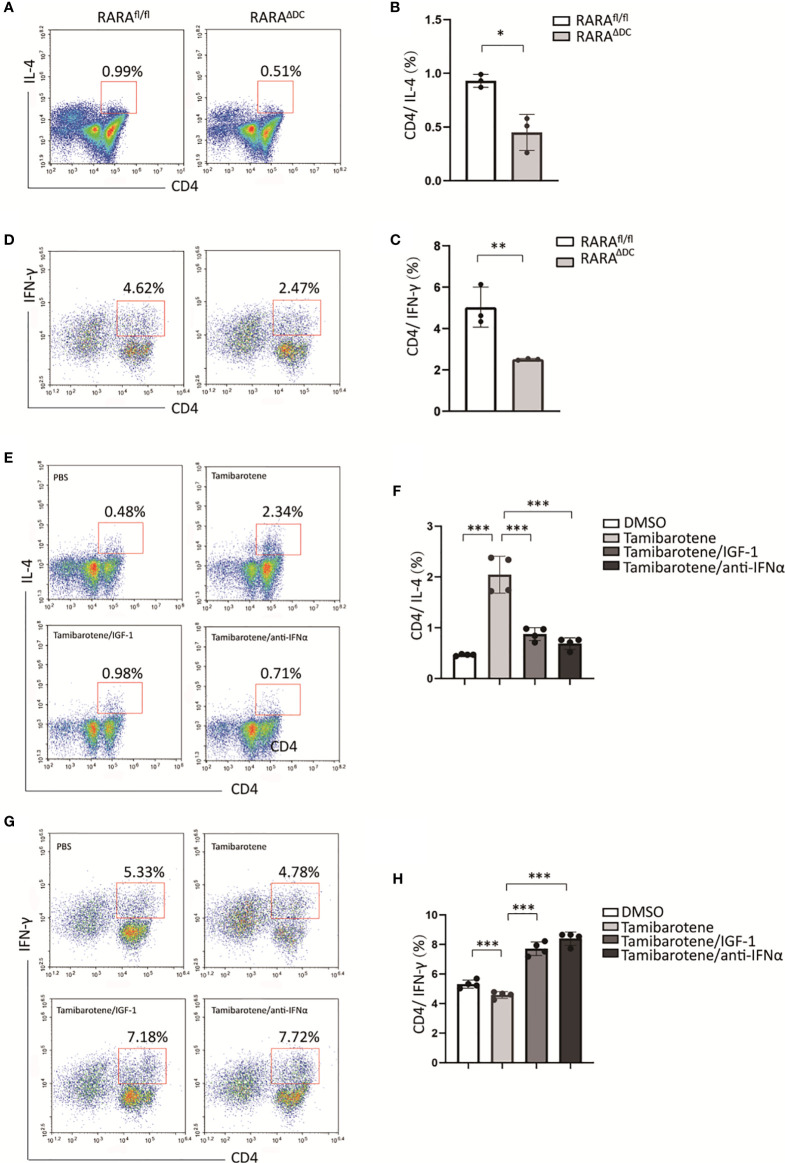
IGF-1 suppresses Th2 differentiation instructed by RARα-activated BMDC. **(A–D)** After BMDCs from RARα^fl/fl^ or RARα^ΔDC^ mice co-cultured with naive T cells isolated from B6 mice, the percentage of CD4(+)IL-4(+) T cells and CD4(+)IFN-γ(+) T cells were detected by flow cytometry.(E-H) BMDCs from B6 mice were treated with DMSO, Tamibarotene, IGF-1 or anti-IFNα, and then co-cultured with naïve T cells. Percentage of CD4(+)IL-4(+) cells and CD4(+)IFN-γ(+)were measured by flow cytometry. Data are from at least two independent experiments. Graph shows mean and SEM. Data were analyzed by unpaired Student’s t-test **(A–D)** and one-way ANOVA **(E–H)**. *p < 0.05, **p < 0.01, ***p < 0.001.

To figure out whether RARα regulates th2 cell differentiation, we next compared the ability of BMDCs from RARA^ΔDC^ mice and which from RARA^fl/fl^control mice instruct T cell responses *in vitro*. The polarization of CD4(+) T cells was detected by flow cytometry. Our results showed that the proportion of CD4(+)IL4(+) T cells stimulated by RARα-deficient BMDCs is significantly smaller than which produced by normal BMDCs (**Figures 5A, B**). Interestingly, RARα-deficient BMDCs also inhibit naive CD4 T cells differentiate to CD4(+)IFN-γ(+) T cells (**Figures 5C, D**). Furthermore, tamibarotene-stimulated BMDCs also promote the proportion of CD4(+)IL4(+) T cells. The previous *in vitro* experiment indicated that IGF-1 could mitigate RA signaling through the Akt pathway. We use IGF-1 to inhibit the tamibarotene’s ability to activate RA signaling. And we found IGF-1 could decrease the number of CD4(+)IL4(+) T cells activated by tamibarotene-stimulated BMDCs (**Figures 5E, F**). And the ability of tamibarotene-stimulated BMDCs to induce Th2-cell differentiation was repressed by anti-IFNα. Collectively, RARα in dendritic cells has a crucial role in instructing Th2 cell differentiation, and IGF-1 can inhibit this effect.

### SCFAs Interfere With Pathological Processes of Food Allergy in ABX-Treated Mice

**Figure 6:**
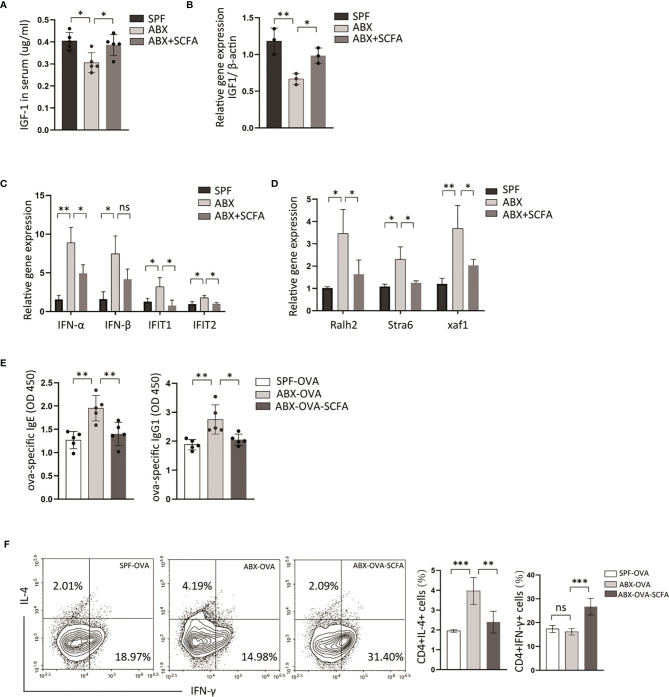
SCFAs alleviate OVA-sensitization fueled by microbiota-dysbiosis. B6 Mice were treated with control vehicle, ABX alone, or with SCFA supplement. **(A)** And then the expression levels of IGF-1 in serum were detected by ELISA. **(B)** The transcriptional levels of IGF-1 in mLN were measured by RT-PCR. The transcriptional levels of type I interferons genes **(C)** and retinoic acid related genes **(D)** in mLN tissue were measured by RT-PCR. Then we established ova-allergic mice model fuled by microbiota dysbiosis with ABX treatment, and SCFAs were used to treat these mice. **(E)** The ova-specific IgE and IgG1 in serum were detected by ELISA. **(F)** The proportions of CD4(+)IL4(+), CD4(+)IFN-γ(+) cell were detected by flow cytometry. Graph shows mean and SEM. Data were analyzed by one-way ANOVA. *p < 0.05, **p < 0.01, ***p < 0.001. ns, not significant.

Some specific metabolites produced by gut microbiota affect host immune function, and emerging evidence indicates that it is involved in the pathogenesis of several diseases (27, 28). SCFAs are the primary microbiota metabolites in the gut. A study demonstrated that supplementation of antibiotic-treated mice with SCFAs restores IGF-1 expression levels in serum and bone. In this experiment, antibiotic depletion of microbiota from normal raised mice reduced serum IGF-1 levels and IGF-1 gene expression in mLN (**Figures 6A, B**). Moreover, IGF-1 levels in serum and mLN are significantly increased in SCFAs-supplemented mice than antibiotic-treated mice (**Figures 6A, B**). Then we measured transcriptional levels of RA signaling-related genes and type I interferons in mLN by qPCR, and both were increased in ABX-treated mice, compared with control mice (**Figures 6C, D**). And we found that after SCFAs supplementation to ABX-treated mice, decreased transcriptional levels of RA-related genes and type I interferons’ genes were detected compared to mice only treated with ABX (**Figures 6C, D**).

To explore whether depletion of microbiota could fuel allergic response, an ova-allergic mice model was established. A higher level of ova-specific IgE, IgG1, was detected by ELISA in antibiotics-treated allergic mice compared with control allergic mice (**Figure 6E**). The antibiotic method also induced an increased number of CD4(+)IL4(+) T cells and a decreased number of CD4(+)IFN-γ(+) T cells in mLN compared with control allergic mice (**Figures 6E, F**). And SCFAs supplementation reduced the ova-specific IgE, ova-specific IgG1 serum levels, and the proportion of CD4(+)IL4(+) T cells compared with antibiotic-treated mice (**Figures 6E, F**). These data indicated that SCFAs could reverse abnormal activation of RA signaling and IFNI response induced by depletion of gut bacteria, and revealed a contribution of RA signaling and type I interferons to intestine allergic response fueled by dysbiotic gut bacteria.

## Discussion

RA, the primary metabolite of vitamin A, serves as the immune regulator and maintains gut homeostasis. Evidence showed that RA secreted mainly from intestinal epithelial and dendritic cells developed to mitigate inflammation in several diseases (29, 30). However, studies recently revealed the proinflammatory aspect of RA signaling, which regulates T cell’s response by affecting IL-17 and IFN-γ secretion (31, 32). RARα, a nuclear receptor, served as the most critical transcriptional modulator in RA signaling. Dysregulated T cells response is involved in allergic diseases. This study clarified RARαplayed a crucial role in food allergy by promoting naive CD4(+) T cells to differentiate into Th2 cells. The gut microbiota has recently proved to be involved in the pathogenesis of several diseases, including allergic diseases, tumors, and even neurological disorders (33, 34). Ablation of gut microbiota early in life could lead to food allergy development by influencing the immune system development (33). In this study, we demonstrated a direct role of gut bacteria in regulation of RA signaling. After ABX treatment to mice, we found an increasing level of RA-related genes and type I interferons (**Figures 6C, D**). We hypothesis that RA signaling and type I interferons are involved in the pathogenesis of food allergy. In an ova-allergic mice model, cd11c^cre^RARA^fl/fl^ mice presented a decreased number of CD4(+)IL4(+) T cells in mLN and reduction of ova-specific IgE, IgG1 level in serum compared with RARA^fl/fl^ control mice (**Figures 1F–H**).

Evidence indicates reducing serum IGF-1 level after ablation of gut microbes (35), resulting in an abnormality of bone formation directly (20). In WT mice, we observed a significant reduction of serum IGF-1 level and IGF-1 expression in mLN after ABX treatment (**Figures 2A, B**). These results indicated IGF-1 might be involved in the regulatory pathway in intestinal diseases associate with microbiota deficiency. In vitro experiment revealed that IGF-1 negatively regulates RA signaling (**Figures 3A, B**) and type I interferons expression (**Figures 4A–E**) activated by tamibarotene RARα specific agonist.

RARα transactivation is activated by RA directly and is also regulated by Phosphorylation, and RARα phosphorylation is proved to contribute to inhibit RA signaling in lung cancers (36). Protein kinase A (PKA), PKC, p38 are considered to phosphorylate retinoic acid receptors (37–39). Another serine/threonine kinase, Akt, is a downstream target of the PI3K pathway and inhibits RARα’s transactivation by phosphorylating the Ser^96^ site of RARα (21). We showed that tamibarotene increased the transcriptional level of RARE element in BMDCs from the RARE report mice (**Figures 3A, B**) and decreased the phosphorylation level of RARα at the Ser^77^ site (**Figures 3C, D**). These results indicated that the phosphorylation of RARα could restrict RA signaling activated by tamibarotene. And it is plausible that Akt, which increases the Phosphorylation of RARα, is involved in the inhibition of RA signaling by IGF-1. Taken together, IGF-1 may inhibit RA signaling by enhancing the process of RARα Phosphorylation by Akt.

IFN-α and IFN-β, the prominent members of Type I interferons, are crucial for the maturation or differentiation of innate immune cells, especially dendritic cells (40). BMDCs from Ifnar1/2-/- mice showed a decreased expression of co-stimulatory factors and a suppressed state of activation compared to wild-type mice (12, 41).

In BMDCs from RARA^ΔDC^ mice, the expression level of IFN-α, IFN-β, and several interferon-stimulated genes was significantly decreased compared with control mice (**Figures 4A–E**). According to these results, we came up with the hypotheses that IGF-1 may regulate type I interferon expression by regulating the RARα pathway. By performing the CHIP experiment in BMDCs from WT mice, we clarified that RARα is bound to the promoter of IFN-α (**Figure 4F**) but not IFN-β. Although the previous study showed RARα targeted stat1 promoter, we did not detect the bond of stat1 and RARα by the CHIP experiment (data not shown). However, we found a decreased stat1 protein level in BMDCs from RARA^ΔDC^ mice compared with those from RARA^fl/fl^ control mice (**Figure 4H**). And RARα agonist enhanced the expression of stat1 protein in a dose-dependent experiment (**Figure 4G**). Hence, we concluded that the expression of type I interferons in BMDCs is affected by the transcriptional level of IFN-α targeted by the transcription factor, RARα.

Consist of animal experiments, RARα-deficient BMDCs significantly inhibit naive CD4 T cells differentiate to CD4(+)IL4(+) T cells (**Figures 5A, B**). And tamibarotene, the RARα specific agonist, enhanced the initiation of Th2 differentiation (**Figures 5E, F**). Interestingly, we found that IGF-1 could mitigate the activation of Th2 differentiation induced by the RARα agonist (**Figures 5E, F**). And we suggested that IGF-1’s inhibition of RARα transcriptional activity and Type I interferon expression might be involved in the inhibition of Th2 cells differentiation of naive T cells *in vitro*. In IFNAR-deficient mice, type 2 immunity was reduced in the absence of IFNI signaling in silica-exposed M. tuberculosis-infected mice (42). Exposure with IFNa increases the sensitivity of T cells to IL-4 and enhances the induction of STAT6 activation (43).

As one of the most studied bacterial metabolites, SCFAs play a core role in mucosal integrity and implicate immune response regulation. In food-allergic children, a decreased level of fecal SCFAs, particularly butyrate, has been described compared to healthy children (44). Acetate, propionate and butyrate are main SCFA, which are substrates for gut bacterial fermentation. In this study, we made a mixture of SCFAs, including acetate, propionate and butyrate. We observed the protective effect in allergic mice after SCFAs supplement. However the specific SCFA for regulation of allergic response should be further studied.

*In vivo* experiment, we demonstrated that SCFAs attenuated ova-allergic responses enhanced by ABX treatment (**Figures 6E–G**). In ABX mice, we observed that the supplement of SCFAs restored decreased IGF-1 expression level in mLN caused by antibiotics treatment (**Figure 6B**). Antibiotics treatment strongly activated RA-related gene expression and type I interferons expression in mLN and suppressed by SCFAs supplement (**Figures 6C, D**). These results provoke the hypothesis that limiting the RA signaling and type I interferons *via* SCFAs could be a mechanism by which gut commensals inhibit allergic response.

In conclusion, we show that gut commensals plays a protective role in the regulation of allergic response in an mice model. Our current work establish a clear link between RA signaling activation in state of dysbiotic community and its effect on type I interferons. However, exact signaling mechanism that SCFAs affect IGF-1 production remains unclear. And the specific microbiota and its mechanism which play a role in state of dysbiotic gut homeostasis fueling allergy should be further determined. Current work identified RARα was involved in regulation of allergic response by driving Th2 cells differentiation of naive CD4 T cells. As IGF-1 inhibits Th2 differentiation of naive T cells by interfering with type I interferons expression in dendritic cells through Akt/RARα pathway, microbiota metabolites SCFAs might attenuate allergic response by maintaining IGF-1 expression. This study reveals that control of RA signaling might be a potential strategy for intervention in allergic diseases in which dysbiotic gut commensals was observed.

## Materials and Methods

### Mice

RARE-lacZ transgenic mice, OT-II transgenic mice, were all on a C57BL/6 background and obtained from Jackson Laboratories. CD11c^cre^, RARα^fl/fl^mice, and C57BL/6 mice were purchased from GemPharmatech. All mice were bred and maintained at the southwest medical university under specific pathogen-free conditions. Experiments were approved from the affiliated hospital of the southwest medical university animal ethics committee and consistent with the NIH guidelines.

### Antibiotics and SCFA Treatment

For antibiotics treatment experiments, mice were bred with antibiotics mixed in pathogens-free water for 2 months. The antibiotics cocktail contained vancomycin (500mg/l), metronidazole (40mg/l), Kanamycin (80mg/l), Gentamycin (7mg/l), and Colistin (9mg/l). For SCFA supplementation experiments, mice were firstly fed with ABX water for two weeks, and then SCFAs (67.5 mM acetate, 40mM butyrate, 25.9mM propionate) were added to ABX water for 1.5 months.

### Food Allergy Mice Model

Mice were intragastrical sensitized with 2mg of ova and 15ug of CT as adjuvant once a week for five weeks. Control mice were treated with PBS only. On day 35, mice were intragastrical challenged with 20mg of mice alone. On day 36, mice were sacrificed for sample collection.

### Cell Culture

For BMDC generation, bone marrow cells obtained from femurs and tibia of adult mice were cultured at 37°C and 5% CO_2_ in RPMI 1640 (Hyclone) containing 10% FBS, 10mM HEPES, 2mM L-glutamine, 50mM 2-mercaptoethanol (Sigma), 50 U/ml penicillin, and 50ug/ml streptomycin. The culture medium was added with cytokines IL4 and GMSCF (PeproTech) on day 2. Day 5 BMDCs were harvest on day seven and exposed to 4mM tamibarotene, 500ng/ml IGF-1, 5ug/ml OVA, respectively, for experiments. For BMDC-T cell co-culture, naive T cells were isolated by CD4(+) negative separation Kit (Stemcells) from OT-II mice and co-cultured with BMDCs at a ratio of 1:2 in the presence of ova for three days. Cells were prepared for flow cytometry analysis.

### Flow Cytometry Analysis

Single-cell suspensions were incubated in ice-cold flow cytometry buffer and stained with fluorochrome-conjugated antibodies purchased from eBioscience: cd11c, b220, cd4, Biolegend. For analysis of secreting cytokines, cells were incubated in the presence of PMA, and Ionomycin for 4h, and 10ug/ml brefeldin A for 2h. BMDCs generated from RARE-lacZ transgenic mice were stimulated with tamibarotene, IGF-1 for 3h and resuspended with DDAOG for 0.5h at 4℃. RA activity was identified by Flow cytometry, measuring the signal of DDAOG to DDAO conversion.

### CHIP

All experimental procedures were instructed by the manufacturer instruction of the CHIP kit (ThermoFisher Scientific). After sonication, cells were broken and immunoprecipitated by anti-RARα (Diagenode). Samples were treated with proteinase K, heated to de-cross linking, and purified with columns. Rabbit IgG was used as a negative control. Immunoprecipitated chromatin was subjected to quantitative PCR analysis with primers targeting the promoters of IFN-α, IFN-β. The two pairs of primers were designed as follows:

IFN-α (sense 1:5’GAAGTTCACCCCAATGATCTG 3’, sense 2:5’GGGCTATAGATGTGCTGAACTG 3’),
IFN-β (sense 1: 5’ TGACTAAGGGCAAAGTGAGATT 3’, sense 2: 5’ TTCACATTCCTTTATTTGGTCAT 3’)

### qPCR

Mesenteric lymph nodes tissues were ground into single cells. Total RNA was extracted using the Total RNA Isolation Kit (Vazymy). And cDNA was synthesized with a cDNA Synthesis Kit (Vazymy). Real-time PCR was performed on LightCycler480 (Roche) using SYBR Green Master Mix (Vazymy). Gene expression was normalized toβ-actin and calculated using the ΔΔCt method.

### Protein Analysis

ELISA detected concentrations of IGF-1 in serum according to the manufacturer’s instruction (Boster). Immunoblot analysis was performed with an antibody to RARα, and RARα phosphorylated at Ser77, antibody to Akt and Akt phosphorylated at Ser473, antibody to stat1, antibody to β-actin (all from Abcam).

### Statistical Analysis

All statistical analyses were performed in Graphpad Prism software (version 8), and data were analyzed by unpaired Student’s t-test and one-way ANOVA whenever necessary. P <0.05 was considered significant. All data represent means ± SEM.

## Data Availability Statement

The raw data supporting the conclusions of this article will be made available by the authors, without undue reservation.

## Ethics Statement

Experiments were approved from the Affiliated Hospital of the Southwest Medical University animal ethics committee and consistent with the NIH guidelines.

## Author Contributions

ZZ conceived the study. XY and ZZ wrote the manuscript. XY, HT, RW, XL, and HJ performed the experiments. ZL helped with manuscript writing and data discussion. All authors contributed to the article and approved the submitted version.

## Funding

This work was supported by the National Natural Science Foundation of China (number 82001703); open funding from the Key Laboratory of Allergy and Immunology, Shenzhen University, and the Shenzhen Science and Technology Peacock Team Project (number KQTD20170331145453160).

## Conflict of Interest

The authors declare that the research was conducted in the absence of any commercial or financial relationships that could be construed as a potential conflict of interest.

## Publisher’s Note

All claims expressed in this article are solely those of the authors and do not necessarily represent those of their affiliated organizations, or those of the publisher, the editors and the reviewers. Any product that may be evaluated in this article, or claim that may be made by its manufacturer, is not guaranteed or endorsed by the publisher.
